# The Devon and Exeter Hospital

**Published:** 1895-03-23

**Authors:** 


### EDITOR'S LETTER-BOX.

[Our correspondents are reminded that prolixity is a great bar to publication, and that brevity of style and conciseness of statement greatly facilitate early insertion.]

### THE DEVON AND EXETER HOSPITAL.

Mr. Charles Cole, architect and surveyor, 50, High Street, Exeter, writes to us as follows: I notice in your issue of The Hospital of the 16th inst., in referring to the contemplated alterations at the above institution, you say that none of the recommendations of Mr. Turner, the architect, were in effect adopted. Allow me to say that I am the architect, and prepared the sketch plans, Mr. Turner was called in to report on the same. I do not know the source of your information, and do not wonder at your querying the amount £65 as the cost of the proposed new sanitary appliances; my approximate estimate of the cost is £650. With regard to the proposed sanitary towers for the Halford wing, the committee certainly consider them most desirable, but on financial grounds, have been compelled to delay their erection.

*** We have received the correction referred to above from another correspondent, and we are glad to learn that the hospital has just received a legacy of £5,000, which will enable the committee to proceed not only with the sanitary towers, to the importance of which they are fully alive, but also with other necessary improvements.—Ed. *T. H.*

